# A fast method to evaluate in a combinatorial manner the synergistic effect of different biostimulants for promoting growth or tolerance against abiotic stress

**DOI:** 10.1186/s13007-022-00943-6

**Published:** 2022-09-15

**Authors:** Patricia Benito, Daniele Ligorio, Javier Bellón, Lynne Yenush, José M. Mulet

**Affiliations:** 1 Instituto de Biología Molecular y Celular de Plantas (IBMCP), Universitat Politècnica de València-Consejo Superior de Investigaciones Científicas, 46022 Valencia, Spain; 2 Caldic Ibérica, S. L. U. Llobateras 23-25, pol.ind. Santiga, 08210 Barberà del Vallés, Barcelona Spain

**Keywords:** Biostimulant, Synergies, *Saccharomyces cerevisiae*, *Arabidopsis thaliana*, Abiotic stress, Growth promoters, Model system, Salinity, Drought

## Abstract

**Background:**

According to the most popular definition, a biostimulant is any substance or microorganism applied to plants with the aim to enhance nutrition efficiency, abiotic stress tolerance and/or crop quality traits, regardless of its nutrient content. Therefore, a biostimulant can help crops to withstand abiotic stress, while maintaining or even increasing productivity. We have previously designed a sequential system, based on two different model organisms, the baker’s yeast *Saccharomyces cerevisiae* and the plant *Arabidopsis thaliana*, to evaluate the potential of different natural extracts as biostimulants employing a blind-test strategy.

**Results:**

In this report, we further expand this concept to evaluate different biostimulants in a combinatorial approach to reveal the potential additive, synergistic or antagonistic effects of different combinations of biostimulants in order to design new formulations with enhanced effects on plant growth or tolerance to abiotic stress. The method is based on yeast assays (growth tests in solid medium, and continuous growth in liquid cultures) and plant assays (mass accumulation in hydroponic culture) to assess effects on early growth.

**Conclusions:**

With this novel approach, we have designed new formulations and quantified the ability to enhance growth and promote biomass accumulation under normal conditions and in the presence of abiotic stresses, such as drought, salinity or cold. This method enables a fast screen of many different products in a combinatorial manner, in order to design novel formulations of natural extracts with biostimulant potential.

**Supplementary Information:**

The online version contains supplementary material available at 10.1186/s13007-022-00943-6.

## Introduction

Modern agriculture is facing many constraints due to anthropogenic global warming, environmental damage from agricultural and industrial production, and the increasing world population. According to the Food and Agriculture Organization of the United Nations (FAO), the estimated world population in 2050 will be around 9 billion inhabitants, 34% higher than today. This means that food production will need to increase around 70–80% [1]. In the twentieth century, the Green Revolution contributed to poverty reduction, prevented hunger for millions of people, and avoided the conversion of thousands of hectares of land into agricultural cultivation [2], but as an adverse side effect, new agricultural practices induced a genetic erosion of agricultural species, release of greenhouse gases, as well as the accumulation of agrochemicals in soils, surfaces and ground waters. The environmental consequences were not caused by the Green Revolution technology itself, but rather by injudicious and overuse of inputs and expansion of cultivation into areas that could not sustain high levels of intensification [3, 4].

Most current agricultural systems are mainly based on the use of different agrochemicals, such as fertilizers and pesticides and different irrigation systems to maintain crop yields. In many cases, the environmental impact of these agricultural practices is very high. One of the major concerns related to arable land overexploitation is the loss of its fertility (desertification). Among the eight threats to soil identified by the European Strategy for Soil Protection (2004), salinity is considered as one of the most important abiotic stresses that limits crop productivity [5]. More than 800 million hectares of land throughout the world are affected by salinity (including both saline and sodic soils) [6]. It is estimated that worldwide, 20% of total cultivated land and 33% of irrigated agricultural land are affected by high salinity. If this trend continues, 50% of arable land would be salinized by 2050 [7]. Excessive soil salinity affects the establishment, development, and growth of plants, resulting in important losses in productivity [8, 9]. This is due to decreased photosynthetic activity, respiration, protein synthesis as well as physiological, metabolic and energetic changes, and disruption of the ionic and osmotic balance [10–12]. Therefore, another much more sustainable Green Revolution is necessary, where these modified practices reduce the environmental impact of agriculture and promote crop productivity. The loss of fertile soil worldwide is causing an increasing interest in the use of biostimulants as an alternative to synthetic fertilizers.

Biostimulants are an emerging category of crop inputs that can improve crop yield under normal and abiotic stress conditions. They have been gaining more attention in recent years, due to their natural origin and their integration among the environmentally friendly tools that can assist in securing high crop yields [13–15]. The European Biostimulants Industry Council (EBIC), which fosters the role of the biostimulant sector in sustainable agricultural production, defines biostimulants as “substance(s) and/or micro-organisms whose function when applied to crops or the rhizosphere is to stimulate natural processes to enhance/benefit nutrient uptake, nutrient efficiency, tolerance to abiotic stress, and crop quality” (EBIC, 2019). Commercially-available biostimulants can be classified into eight different categories: (1) humic substances, (2) complex organic materials, (3) beneficial chemical elements, (4) inorganic salts, (5) seaweed extracts and botanicals, (6) chitin and chitosan derivatives, (7) anti-transpirants and (8) free amino acids and other nitrogen-containing substances. Living-based products containing beneficial rhizosphere microorganisms, such as plant growth-promoting bacteria (PGPBs) and mycorrhizal fungi, are considered the ninth category [13]. Biostimulants are not considered agrochemicals because they only influence the vigor of plants and do not have a direct action against pests or diseases, nor do they provide nutrients directly to plants [16].

The application of combined biostimulant products from different natural extracts is unusual. The combination of two or more biostimulants can lead to additive, synergistic or antagonistic interactions. Moreover, the combined effect can be different from that obtained by each biostimulant individually [17]. Giordano et al. [18] showed that the total yield and dry biomass of *Diplotaxis tenuifolia* plants treated with a combination of three biostimulants increased on average 48.1% and 37.2% respectively, as well as a leading to higher contents of chlorophyll, potassium, magnesium, and calcium, as compared to the untreated plants. The demand for biostimulants is likely to increase in the coming years for both conventional and organic and low-input agriculture. For this reason, interest in developing a new generation of plant biostimulants for sustainable agriculture from different natural extracts with synergistic interactions is increasing. In addition, there is a growing need for novel products able to maintain yield in the conditions imposed by anthropogenic global warming.

Currently, there are few methods in the literature to evaluate the biostimulant potential of natural extract combinations. Validating the efficiency of natural extract combinations directly in field experiments with crops could be very difficult, due to time, infrastructure, and budget limitations. The aim of this work is to design a novel methodology to evaluate the effect of the combination of several biostimulants. The system is based on the use of two model organisms: *Saccharomyces cerevisiae* and *Arabidopsis thaliana* in laboratory and greenhouse conditions. We have previously shown that the use of a model organism is a cost-effective system to test biostimulant activity in a fast and reliable manner [19]. In this report, we further expand this concept to design a novel system to evaluate synergies between combinations of biostimulants in order to be able to provide new and improved formulations to farmers.

## Material and methods

### Candidate biostimulants

To design the methodology, we tested 10 natural extracts (non-microbial) individually and/or in combination to assess their biostimulant effect. All products were provided by Caldic Ibérica S.L.U (Barcelona, Spain). All products are derived from natural extracts and their description can be found in Table 1. The experiments were performed as blind tests, therefore product composition was unbeknownst to experimenters during the period in which the assays were performed. Stock solutions were made from each individual product at a concentration of 10 mg/ml (w/v). Each stock solution was sterilized by tyndallization.

**Table 1:** Identity of the tested natural extracts

Biostimulant compound number	Description
B1	Tannins extract
B2	Yeast extract
B3	*Ascophyllum nodosum* extract
B4	Licorice root extract
B5	White willow extract
B6	*Pueraria lobata* extract
B7	Dried sage extract
B8	Yucca dry extract (low saponin)
B9	Yucca dry extract (medium saponin)
B10	Quillaja dry extract

### Experimental design

The methodology described in this report is based on the protocol described in Saporta et al. [19] using two different biological model systems sequentially: the brewer's yeast *S. cerevisiae* and the model plant *A. thaliana*. We designed a combinatorial approach based on this confirmed method.

**Fig. 1:**
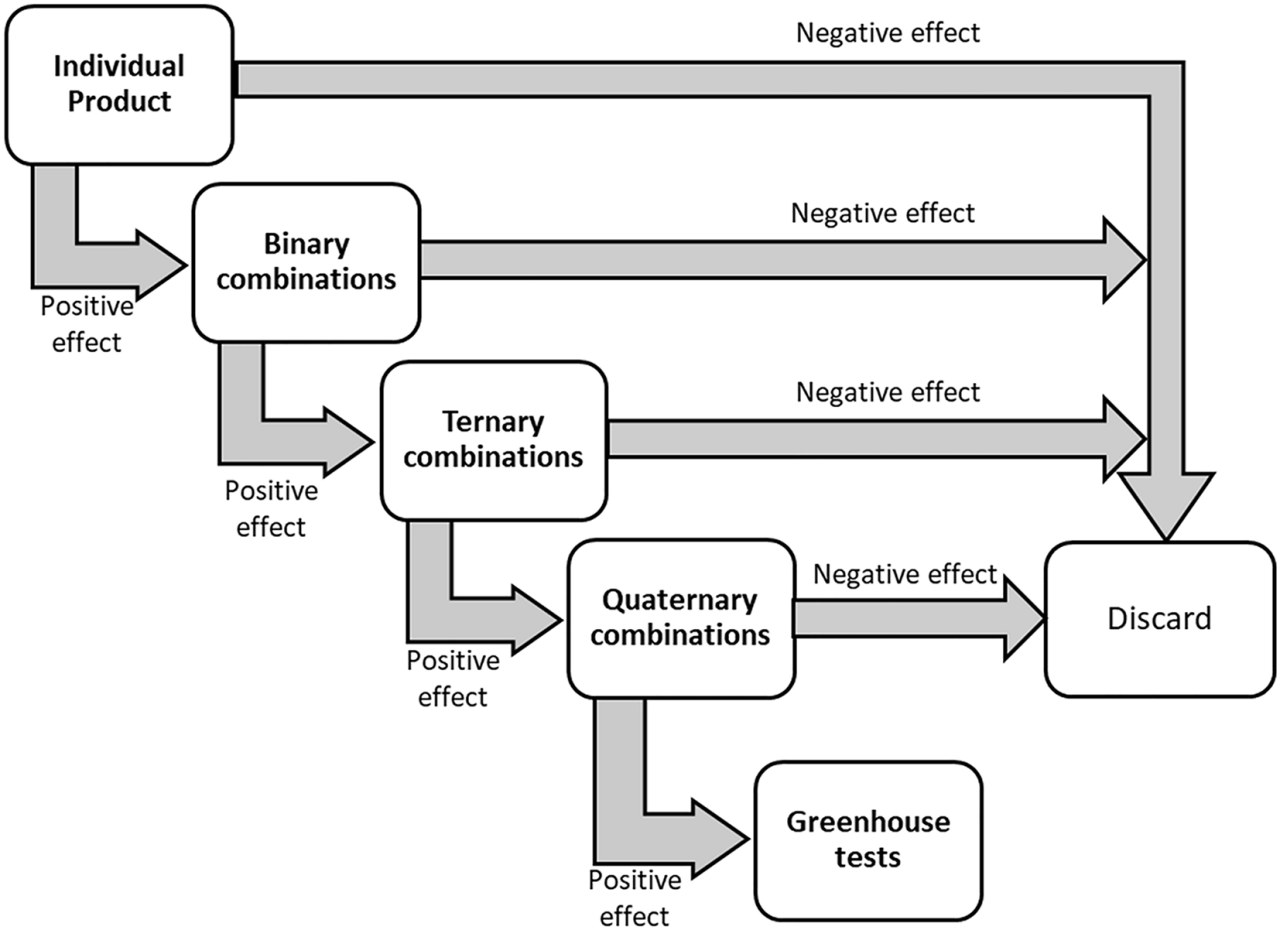
Schematic representation of the evaluation process and choice of the different natural product combinations with biostimulant effect under abiotic stress conditions

Product combinations were made as follows. The biostimulant effect of each individual compound was evaluated under normal and stress conditions by using a previously determined dose of each product. We selected the highest dose in which we did not observe any deleterious effect under normal growth conditions. The natural compounds with the best biostimulant effect were chosen to perform binary combinations with the rest of the compounds. Subsequently, those binary combinations with synergistic effects were chosen, which were again combined with the rest of the compounds to make ternary combinations, discarding those combinations without synergistic effects. The process was repeated until quaternary combinations were obtained, that is, a formulation of four different compounds. A workflow of the method can be found in Fig. 1.

### Yeast growth assays

The growth assays on solid media (drop tests) were performed to determine the toxic dose of natural extracts under normal conditions and the possible biostimulant effect on yeast growth under normal or stress conditions (osmotic, saline and temperature stress). The drop test assays were performed as indicated in Ríos et al. [20]. All assays were performed in solid Yeast extract Peptone Dextrose (YPD) medium (1% yeast extract, 2% bacteriological peptone, 2% glucose, and 2% agar in distilled water). The products assayed, previously sterilized by tyndallization, were added to the medium after autoclaving, but before gelification. Drop tests were performed by growing the wild-type *S. cerevisiae* strain BY4741 (MATa *his3Δ1 leu2Δ0 met15Δ0 ura3Δ0*) [21] cells until saturation in YPD broth. Cell cultures were then diluted 1:10, 1:100, and 1:1000, and spotted onto plates of YPD medium containing 1 M NaCl, 0.15 M LiCl, or 1.8 M Sorbitol, in addition to plates with medium without stress. Yeast was grown for three or four days at 28 °C. Cold and heat stress was applied by growth at 10 °C for four weeks or 37 °C for three days, respectively. Three independent complete experiments and three biological replicates per plate were performed with similar results.

### Continuous growth assays

To test the effect of the assayed products on yeast continuous growth we used *BioScreen*^®^ assays, in which we can follow the growth of each strain and treatment in real-time conditions. Yeast liquid cultures were grown until saturation in YPD broth, then diluted to an initial optical density at 600 nm (OD_600_) of 0.01 in YPD containing the indicated biostimulant product and stress (0.8 M NaCl, 0.15 M LiCl or 1.8 M Sorbitol). Growth was monitored in 100-well Honeycomb Plates using the BioScreen^®^ C microbiological workstation (Oy Growth Curves Ab Ltd., Helsinki, Finland), with automatic recording of OD_600_ every 30 min. The plates were incubated for three days at 28 °C with shaking for 10 s before measuring the optical density. Each point represents the mean of three replicates, with SE < 2% in most cases (error bars are not shown for clarity). From the OD_600_ data over time, the growth rates and generation time were calculated [22]. The experiment was reproduced independently three times with similar results.

### *Arabidopsis* early stages of plant growth assays

A detailed description of plant assays can be found in [23]. For in-vitro culture, seeds of *A. thaliana* wild-type (ecotype Columbia-0) were surface-sterilized with commercial bleach diluted 1:1 (v/v) for 15 min and rinsed with sterile water. Stratification was performed over three days at 4 °C. To measure the effect of biostimulants on the early growth of *Arabidopsis*, the MS medium was prepared without phytoagar, and plants were grown in six-well Cellstar plates (Greiner). The MS medium contained Murashige and Skoog (MS) basal salt mixture (0.22%; Duchefa Biochemie B·V, Haarlem, The Netherlands), sucrose (1%), and 2.6 mM MES (2-(*N*-morpholino) ethanesulfonic acid) buffer, adjusted to pH 5.9 with potassium hydroxide. Each well contained 7 mL of the medium, and three seedlings that had been previously cultivated for six days in an MS solid medium containing 0.8% phytoagar, without additives, and then transferred to the liquid medium with the indicated additive (stress and/or biostimulant; Additional file 1: Fig. S1). The stress conditions used were 140 mM NaCl or 24 mM LiCl for salt stress and 280 mM mannitol for osmotic stress. The plates were grown under long-day chamber conditions (16 h light/8 h dark, 23 °C, 130 μE/m^2^ s, 70% relative humidity) with gentle shaking (100 rev/min). Fresh and dry weight, percentage of flowering plants and water content were recorded in plantlets for 20 days. The water content (WC) was calculated using the following formula: (FW-DW)/DW, where FW is the fresh weight and DW is the dry weight and expressed as gH_2_O/g DW.

### Statistical analysis

Student's test was performed using R software (ver 4.2.1; http://www.R-project.org). These tests were calculated with respect to the increase or decrease in plant biomass compared to the control. The means are considered to be significantly different at *p* < 0.05.

## Results

The use of model organisms *S. cerevisiae* and *A. thaliana* under laboratory conditions can speed up the screening of the biostimulant effect of natural extracts. We wanted to test whether this system could be used to discover interactions among different natural extracts. For this, we tested the individual effect of each extract. Culture plates with serial concentrations of each product were prepared (concentrations from 2000 to 20 µg/ml (v/v)). Once the maximum doses that did not present any observable toxic effect were obtained, the biostimulant effect of the individual or combined compounds was evaluated in yeast by drop test assays under control or abiotic stress conditions (osmotic, saline and cold/heat stress). Subsequently, the biostimulant effect of each product was also evaluated individually or in combination on the yeast growth curve under normal conditions and abiotic stresses in liquid culture. Once the screening in yeast was completed, in vitro plant tests were carried out to evaluate the effect of each extract individually or the combination of different products on early stages of plant growth under normal and abiotic stress conditions. A scheme of the experimental set-up is shown in Fig. 1.

### Determination of the toxic dose of individual products in yeast

First, we determined the toxic dose of each product and the possible biostimulant effect on yeast growth without stress by drop test assays. For this purpose, YPD agar plates were prepared with serial concentrations of each product, ranging from 2000 to 20 µg/ml (v/v). The stock solutions of each assayed compound were prepared at a concentration of 10 mg/ml (w/v). The description of each product can be found in Table 1. These assays were performed under normal growth conditions without abiotic stress. Three biological and technical replications were assayed per natural extract and concentration. A representative figure of the drop test assays can be found in Additional 2: Fig. S2. The results showed that yeast growth was optimal (compared to the control plate without any additive and stress) at the concentration of 800 µg/ml in most of the products tested, except for products B8 and B9, in which optimal yeast growth was observed at a concentration of 200 µg/ml. Neither of the products potentiated yeast growth compared to the control medium without additives. The concentrations obtained were applied in subsequent experiments.

Once we determined the maximum concentration without toxic effect and confirmed that none of the products was enhancing growth under normal conditions, we tested whether the products had a biostimulant effect under abiotic stress. Drop test experiments were performed in YPD agar plates with three different stressors independently: 1 M NaCl, 0.15 M LiCl and 1.8 M sorbitol. NaCl and LiCl exert saline stress, while sorbitol induces osmotic stress. Cold and heat stress also were tested by growing the plates at 10 °C and 37 °C, respectively. The individual evaluation of the 10 natural extracts showed that the three products with stronger effects were B3, B4 and B5 (Table 2).

**Table 2:** Effect of the natural products individually tested under control conditions (YPD without stress), salt (NaCl and LiCl), osmotic (Sorbitol), and temperature (10 °C and 37 °C) stress

	No-stress	NaCl 1 M	LiCl 0.15 M	Sorbitol 1.8 M	37 °C	10 °C
B1	=	=	=	=	−	=
B2	=	=	=	+	=	=
B3	=	=	=	+	=	=
B4	=	+	+	+	=	=
B5	=	+	+	+	=	=
B6	=	=	W	+	W	=
B7	=	=	=	+	=	=
B8	=	−	−	−	=	−
B9	=	−	−	−	−	−
B10	=	W	=	−	=	=

Yeast growth in fresh YPD medium containing 800 µg/ml of each product, except for B8 and B9, of which was used 200 µg/ml. Three independent complete experiments, each using three biological and three technical replicates per plate, were performed with similar results

( +) Growth stimulation; ( =) no observable effect; (−) negative; (W) weak

### Effect of the combination of different natural products with a biostimulant effect on yeast

We then examined the effect of the binary combinations in yeast and observed that product B3 showed a better effect in combination with other extracts. When combined with the compounds B2-through B7, it showed a stimulating effect on yeast growth in the presence of abiotic stress (Table 3). B2 also showed considerable stimulating effects in the presence of several stresses, especially sorbitol. Therefore, compounds B2 and B3 were used as the cornerstone in the different ternary combinations. Different combinations with compounds B1, B2, B3, B4 and B5 displayed a potential synergistic effect (Table 4). Concerning the quaternary combinations, the combination B2–B3–B4–B5 showed the greatest stimulating effect on yeast growth in the presence of saline (1 M NaCl and 0.15 M LiCl) and osmotic (1.8 M Sorbitol) stress (Table 5 and Additional file 2: Fig. S2). Compounds B8 and B9 showed an antagonistic effect when combined with most of the target products (Tables 2, 3, 4, 5).

**Table 3:**
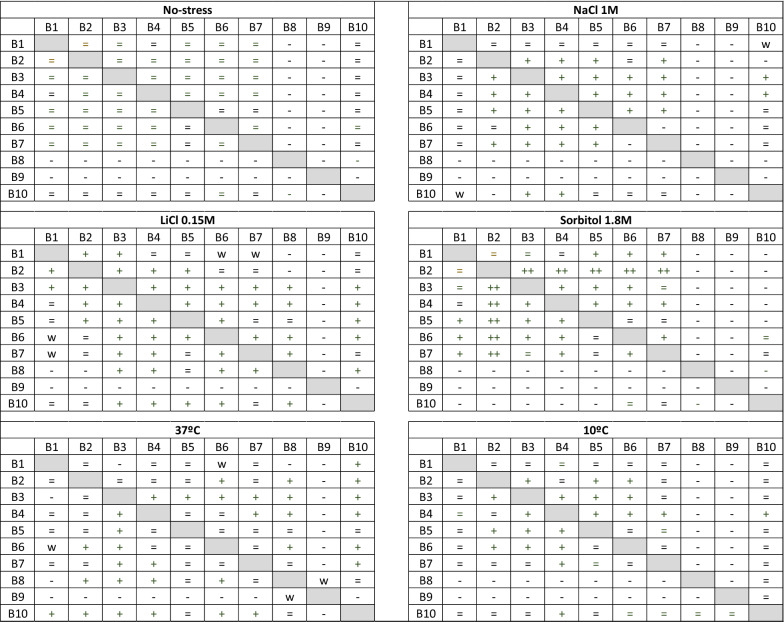
Effect of binary combinations of plant extracts on *S. cerevisiae* under control conditions (YPD without stress), saline (NaCl and LiCl), osmotic (Sorbitol) and temperature (10 °C and 37 °C) stress.

**Table 4:** Effect of ternary combinations of natural extracts on yeast in the presence of control conditions (YPD without stress), saline (NaCl and LiCl), osmotic (Sorbitol) and temperature (10 °C and 37 °C) stress

	No-stress	NaCl 1 M	LiCl 0.15 M	Sorbitol 1.8 M	37 °C	10 °C
B2–B3	=	+	+	=	=	=
B1–B2–B3	=	+	+	+	=	=
B2–B3–B4	=	+	+	++	=	=
B2–B3–B5	=	+	+	+	=	=
B2–B3–B6	=	+	+	+	=	=
B2–B3–B7	=	+	+	+	=	=
B2–B3–B8	−	−	−	−	=	=
B2–B3–B9	−	−	−	−	=	−
B2–B3–B10	=	+	+	−	=	−
B2–B5	=	+	+	++	=	+
B1–B2–B5	=	+	+	+	−	−
B2–B3–B5	=	+	+	+	W	=
B2–B4–B5	=	+	=	+	−	=
B2–B5–B6	=	=	=	+	=	=
B2–B5–B7	=	+	+	+	−	=
B2–B5–B8	=	=	=	=	−	−
B2–B5–B9	−	−	−	−	−	−
B2–B5–B10	=	+	=	=	W	=
B3–B4	=	+	+	++	=	=
B1–B3–B4	=	+	+	++	=	=
B2–B3–B4	=	+	+	++	=	=
B3–B4–B5	=	+	+	+	=	=
B3–B4–B6	=	+	=	++	=	=
B3–B4–B7	=	+	+	++	=	=
B3–B4–B8	−	−	=	−	=	−
B3–B4–B9	−	−	−	−	=	−
B3–B4–B10	=	+	=	+	=	=

In ternary combinations, *S. cerevisiae* was grown in a fresh YPD medium containing 400 µg/ml of each product, except for B8 and B9, which used 100 µg/ml. Three independent complete experiments, each using three biological and three technical replicates per plate, were performed with similar results

(++) High growth stimulation; (+) Growth stimulation; (=) no observable effect; (−) negative; (W) weak

**Table 5:** Effect of quaternary combinations of natural extracts on *S. cerevisiae* growth under control conditions (YPD without stress), saline (NaCl and LiCl), osmotic (Sorbitol) and temperature (10 °C and 37 °C) stress

	No-stress	NaCl 1 M	LiCl 0.15 M	Sorbitol 1.8 M	37 °C	10 °C
B2–B3–B4	=	+	+	=	=	=
B1–B2–B3–B4	=	=	=	=	=	=
B2–B3–B4–B5	=	+	+	+	=	+
B2–B3–B4–B6	=	=	=	=	=	=
B2–B3–B4–B7	=	=	=	=	=	=
B2–B3–B4–B8	−	−	−	−	−	−
B2–B3–B4–B9	−	−	−	−	−	−
B2–B3–B4–B10	=	−	W	W	=	=
B3–B4–B5	=	=	+	=	=	=
B1–B3–B4–B5	=	W	+	=	=	=
B3–B4–B5–B6	=	=	=	=	=	=
B3–B4–B5–B7	=	=	=	=	=	=
B3–B4–B5–B8	−	−	=	−	−	−
B3–B4–B5–B9	−	−	−	−	−	−
B3–B4–B5–B10	=	=	+	W	=	=

In quaternary combinations, 200 µg/ml of each product were used, except for B8 and B9, which used 50 µg/ml

Three independent complete experiments, each using three biological and three technical replicates per plate, were performed with similar results

(+) Growth stimulation; (=) no observable effect; (−) negative; (W) weak

The concentration of each product was 800 µg/ml except for B8 and B9, of which was used 200 µg/ml of final concentration. Three independent complete experiments, each using three biological and three technical replicates per plate, were performed with similar results. (++) Growth stimulation; (+) positive; (−) negative; (W) weak. (NaCl and LiCl), osmotic (Sorbitol) and temperature (10 °C and 37 °C) stress. (++) High growth stimulation; (+) Growth stimulation; (=) no observable effect; (−) negative; (W) weak.

### Evaluation of the biostimulant effect of the different combinations of products in yeast under continuous growth

The analysis of the effect of natural products under continuous growth of *S. cerevisiae* provided quantitative data on the influence of the products on the growth curve. For this, the increase in the turbidity of the cultures was monitored at an optical density of 600 nm every 30 min for three days using a BioScreenC^®^ spectrophotometer (Oy Growth Curves Ab Ltd., Helsinki, Finland). During the different analyses, the concentration of the tested products was adjusted until the most optimal yields were obtained.

**Fig. 2:**
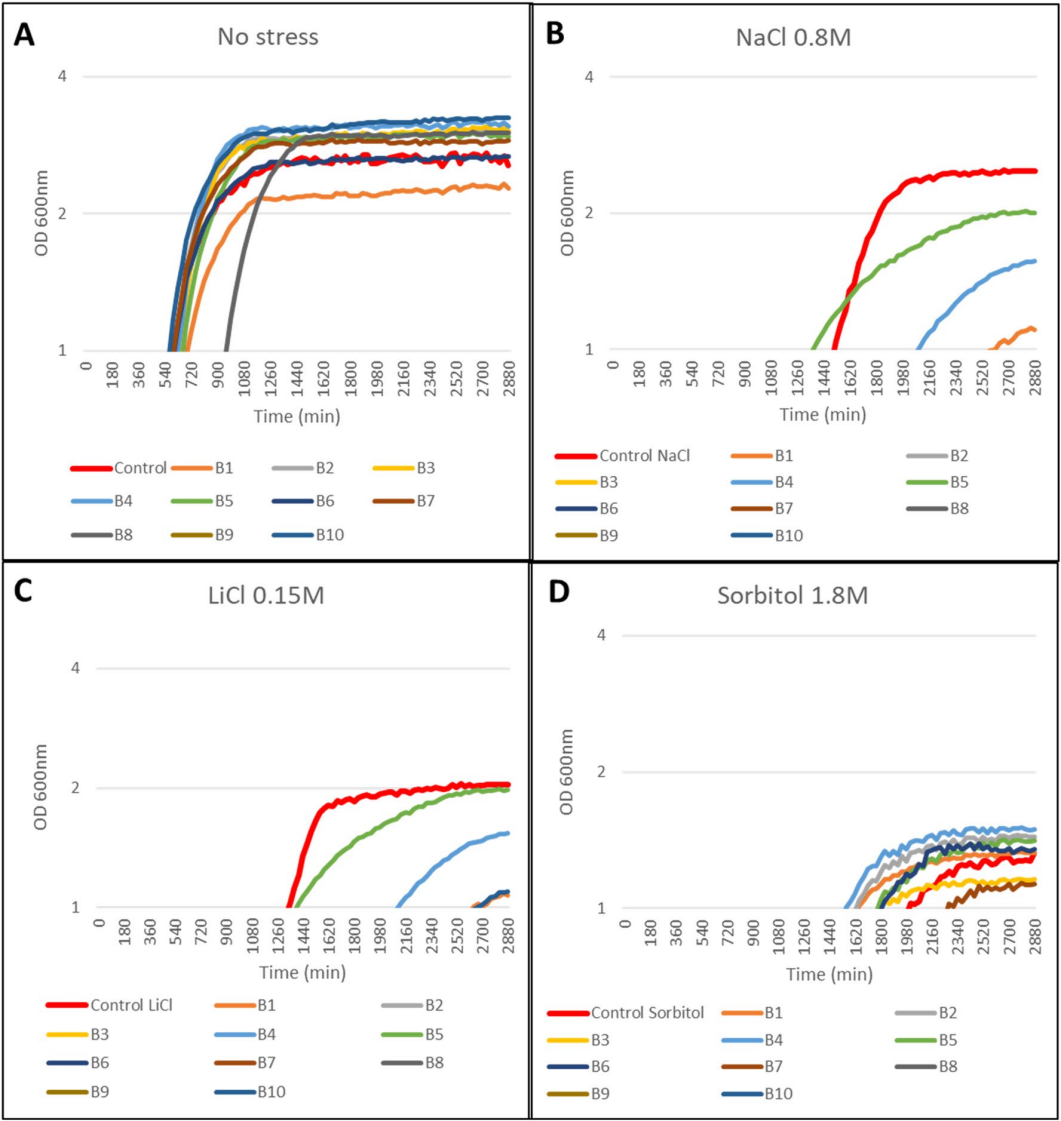
The semi-logarithmic plots of the biostimulant effect of natural extracts individually on the continuous growth of *S. cerevisiae* under **A** control conditions (YPD without stress), and abiotic stress conditions: **B** NaCl 0.8 M, **C** LiCl 0.15 M, and **D** Sorbitol 1.8 M. Liquid cell cultures were grown until saturation in liquid YPD medium, then diluted to an initial OD_600_ of 0.01 in fresh YPD medium containing 800 µg/ml except for B8 and B9, which were used at 200 ug/ml. Growth monitoring was performed by automatic recording of OD_600_ every 30 min. The X-axis represents time in minutes (min) and the Y-axis represents the optical density at 600 nm (OD 600 nm) on logarithmic scale. Each point shows the mean of three replicates, with a standard error < 5% in most cases (error bars not shown for clarity)

**Fig. 3:**
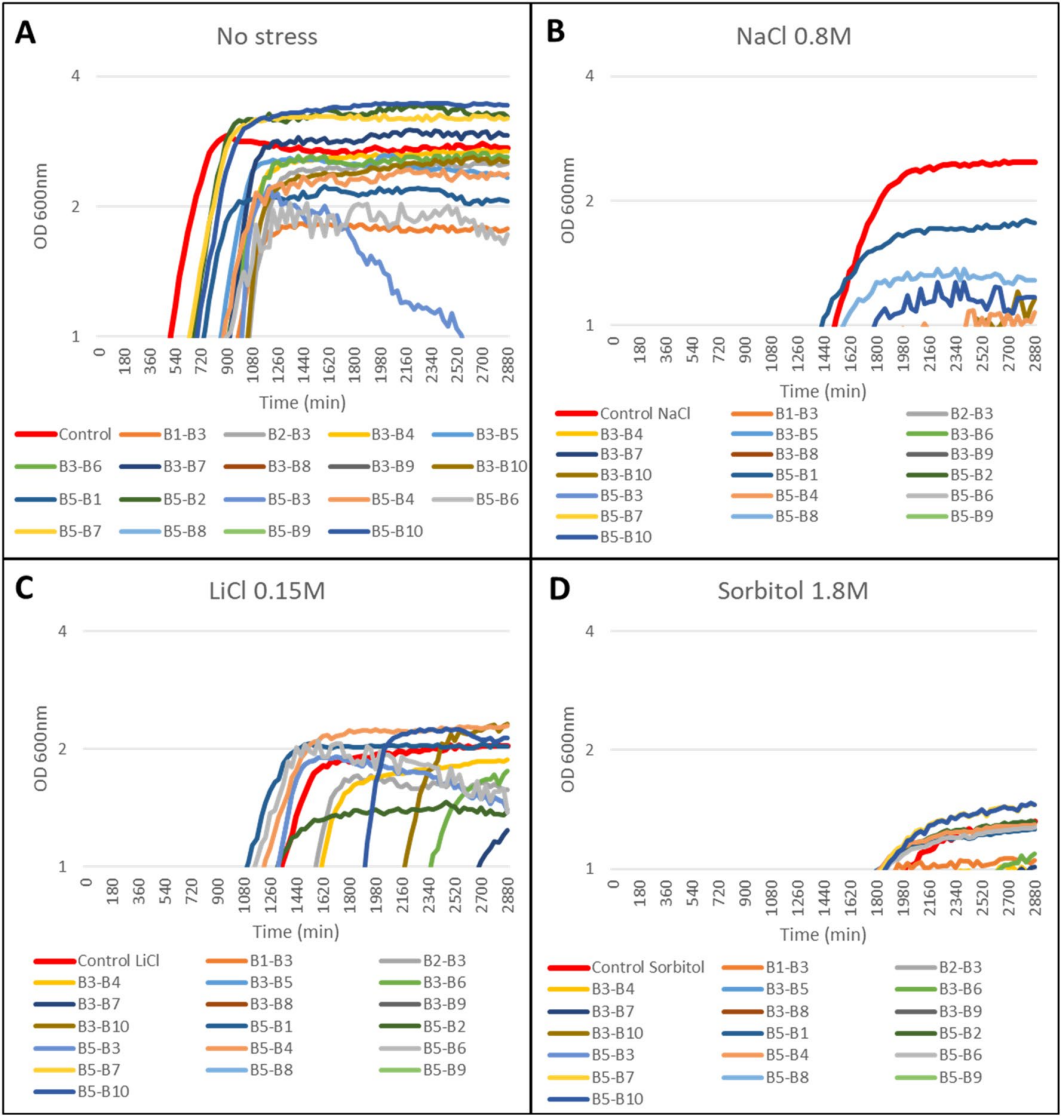
The semi-logarithmic plots of binary combinations on the continuous growth of *S. cerevisiae* under **A** control conditions (YPD without stress), and abiotic stress conditions: **B** NaCl 0.8 M, **C** LiCl 0.15 M, and **D** Sorbitol 1.8 M. Liquid cell cultures were grown until saturation in liquid YPD medium, then diluted to an initial OD_600_ of 0.01 in fresh YPD medium containing 500 µg/ml except for B8 and B9, which were used at 150 µg/ml. Growth monitoring was performed by automatic recording of OD_600_ every 30 min. The X-axis represents time in minutes (min) and the Y-axis represents the optical density at 600 nm (OD 600 nm) on logarithmic scale. Each point shows the mean of three replicates, with a standard error < 5% in most cases (error bars not shown for clarity)

**Fig. 4:**
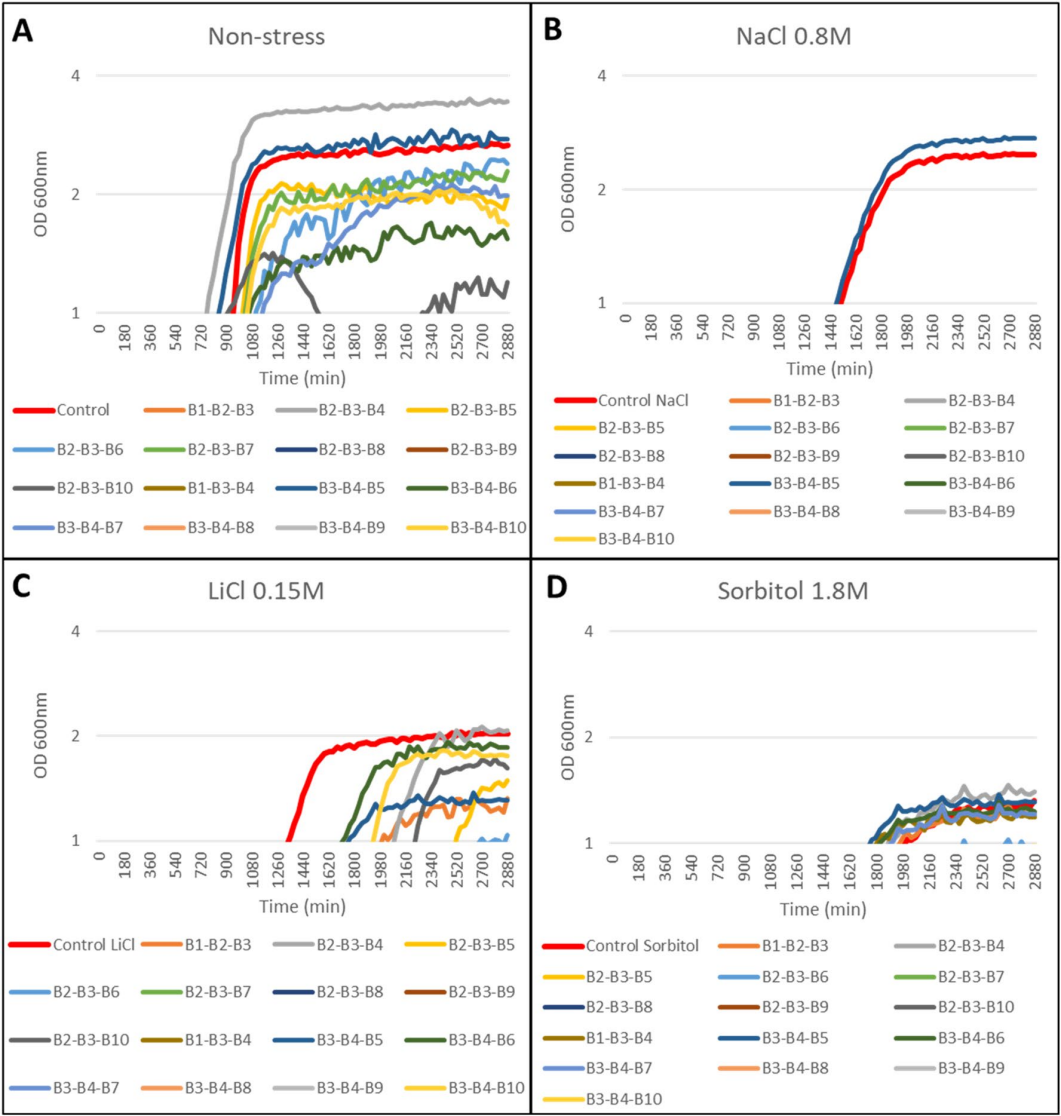
The semi-logarithmic plots of ternary combinations on the continuous growth of *S. cerevisiae* under **A** control conditions (YPD without stress), and abiotic stress conditions: **B** NaCl 0.8 M, **C** LiCl 0.15 M, and **D** Sorbitol 1.8 M. Liquid cell cultures were grown until saturation in liquid YPD medium, then diluted to an initial OD_600_ of 0.01 in fresh YPD medium containing 400 µg/ml except for B8 and B9, which were used at 100 µg/ml. Growth monitoring was performed by automatic recording of OD_600_ every 30 min. The X-axis represents time in minutes (min) and the Y-axis represents the optical density at 600 nm (OD 600 nm) on logarithmic scale. Each point shows the mean of three replicates, with a standard error < 5% in most cases (error bars not shown for clarity)

**Fig. 5:**
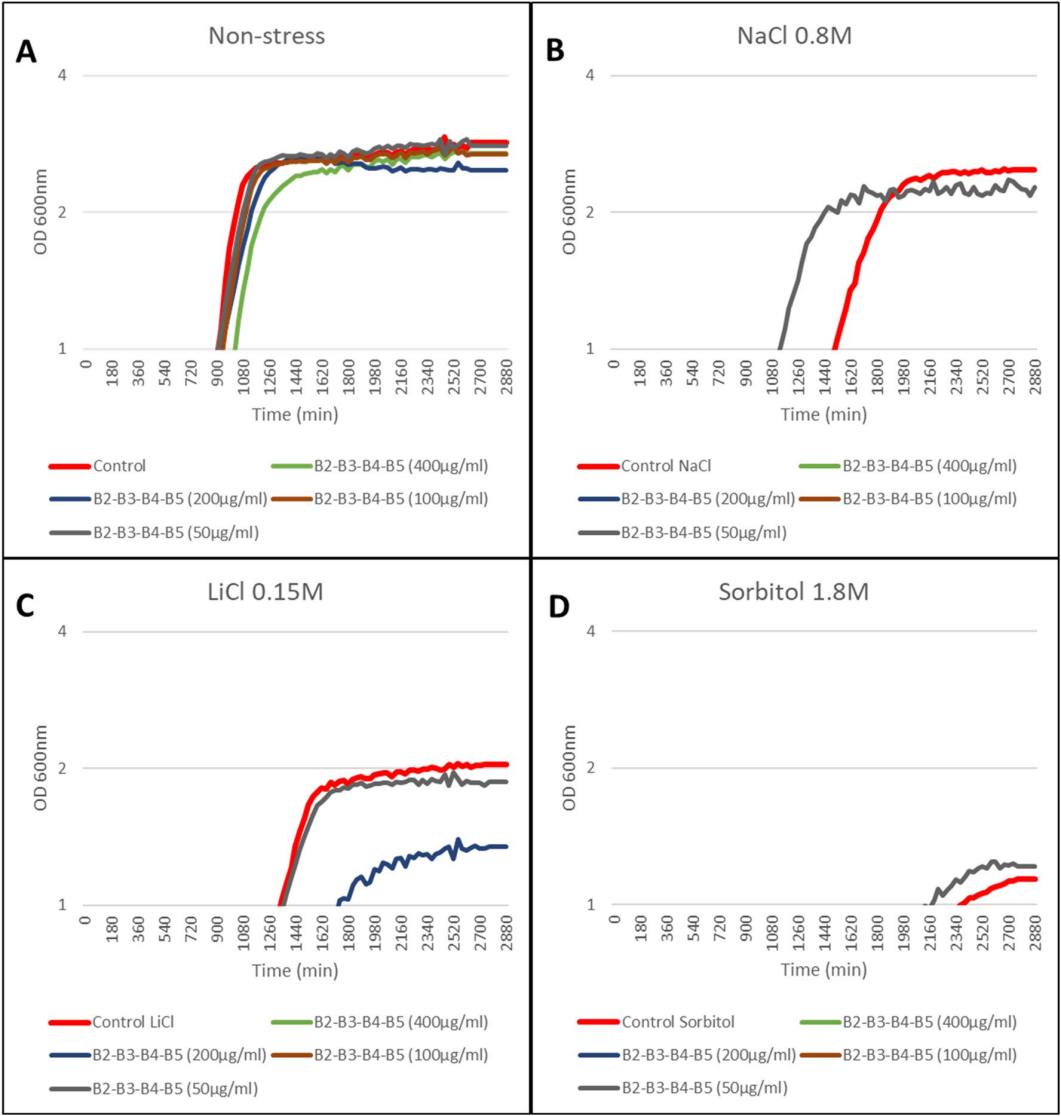
The semi-logarithmic plots of quaternary combinations on the continuous growth of *S. cerevisiae* under **A** control conditions (YPD without stress), and abiotic stress conditions: **B** NaCl 0.8 M, **C** LiCl 0.15 M, and **D** Sorbitol 1.8 M. Different concentrations of natural products were tested to obtain the optimal concentration for the combination of four compounds: 400, 200, 100 and 50 µg/ml of each compound. Growth monitoring was performed by automatic recording of OD_600_ every 30 min. The X-axis represents time in minutes (min) and the Y-axis represents the optical density at 600 nm (OD 600 nm) on logarithmic scale. Each point shows the mean of three replicates, with a standard error < 5% in most cases (error bars not shown for clarity)

Under normal growth conditions (YPD without stress), the products B2, B3, B4, B5, and B10 induced a faster yeast growth and higher yield with respect to the control sample (Fig. 2A), with B5 displaying the best results under salt stress conditions (0.8 M NaCl and 0.15 M LiCl; Fig. 2B, C). In the presence of 1.8 M sorbitol, B2 and B4 were the products with the highest stimulatory effect on yeast growth (Fig. 2D). We then studied binary combinations. B2–B5, B5–B7 and B5–B10 showed a yield average of 117, 110 and 125% respectively under normal conditions (Fig. 3A). B5–B1 showed the best results in the presence of 0.8 M NaCl with respect to the different combinations tested, but both its yield and growth rate were much lower than the control (Fig. 3B). The combination B5–B4, together with B5–B1 and B2–B3, also showed a yield of approximately 113% in the presence of 0.15 M LiCl, while B5–B10 and B3–B10 displayed the same yield but required 100% longer time to reach the exponential phase (Fig. 3C). B2–B3, B5–B7 and B5–B10 displayed the best growth rates under osmotic stress (1.8 M sorbitol; Fig. 3D), as well as under normal growth conditions (Fig. 3A). Among the ternary combinations evaluated, the combinations of the products B2–B3–B4 and B3–B4–B5 showed a yield of around 112% with respect to the control without additives and under salt (NaCl and LiCl) and osmotic (sorbitol) stress (Fig. 4). Finally, the quaternary combination that displayed the best performance was B2–B3–B4–B5, whose components were added to the medium at a concentration of 50 µg/ml (Fig. 5). At this stage, despite the negative effects in the different combinations that include B8 and B9, we undertook the study in the plant model system *A. thaliana* with all the tested products to perform a better validation of the proposed system.

### Effect of natural extracts on the early growth of *A. thaliana*

First, we determined the maximum dose without toxic effects for the model plant *A. thaliana*. We used the wild-type Columbia 0 ecotype. For this, six-well plates were used with 7 ml of MS medium (without abiotic stress) with different product concentrations from 800 to 20 µg/ml (v/v) from stock solutions of each compound at a concentration of 10 mg/ml (w/v). Biomass accumulation was determined by measuring the fresh and dry weight after 3 weeks. Three biological and technical replications were performed per natural extract and concentration. Most of the compounds showed a maximum non-toxic dose at a concentration of 100 µg/ml, except B6 and B7, which showed a non-toxic dose of 200 µg/ml, and B2 at 400 µg/ml.

**Fig. 6:**
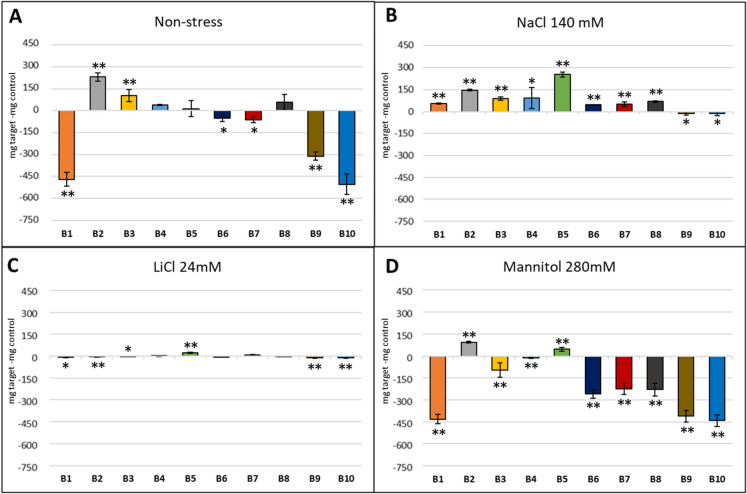
Effect of individual compounds on fresh weight of *A. thaliana* Col-0 WT under **A** control conditions (MS without stress) and in the presence of abiotic stress conditions: **B** NaCl 140 mM, **C** LiCl 24 mM, and **D** mannitol 280 mM. The optimal concentration of the natural extracts in *Arabidopsis* early growth was a concentration of 400 µg/ml (v/v) for B2 and B8, while the rest of the products showed an optimal concentration of 200 µg/ml (v/v). The X-axis indicates the tested product while the Y-axis represents the difference in fresh weight compared to the control sample without biostimulant. Statistical data from three experiments (n = 30 for each experiment) are presented. The bars represent a standard error. *p < 0.05 by Student’s tests for increased early development compared to the control; **p < 0.01 by Student’s tests for increased early development compared to the control

**Fig. 7:**
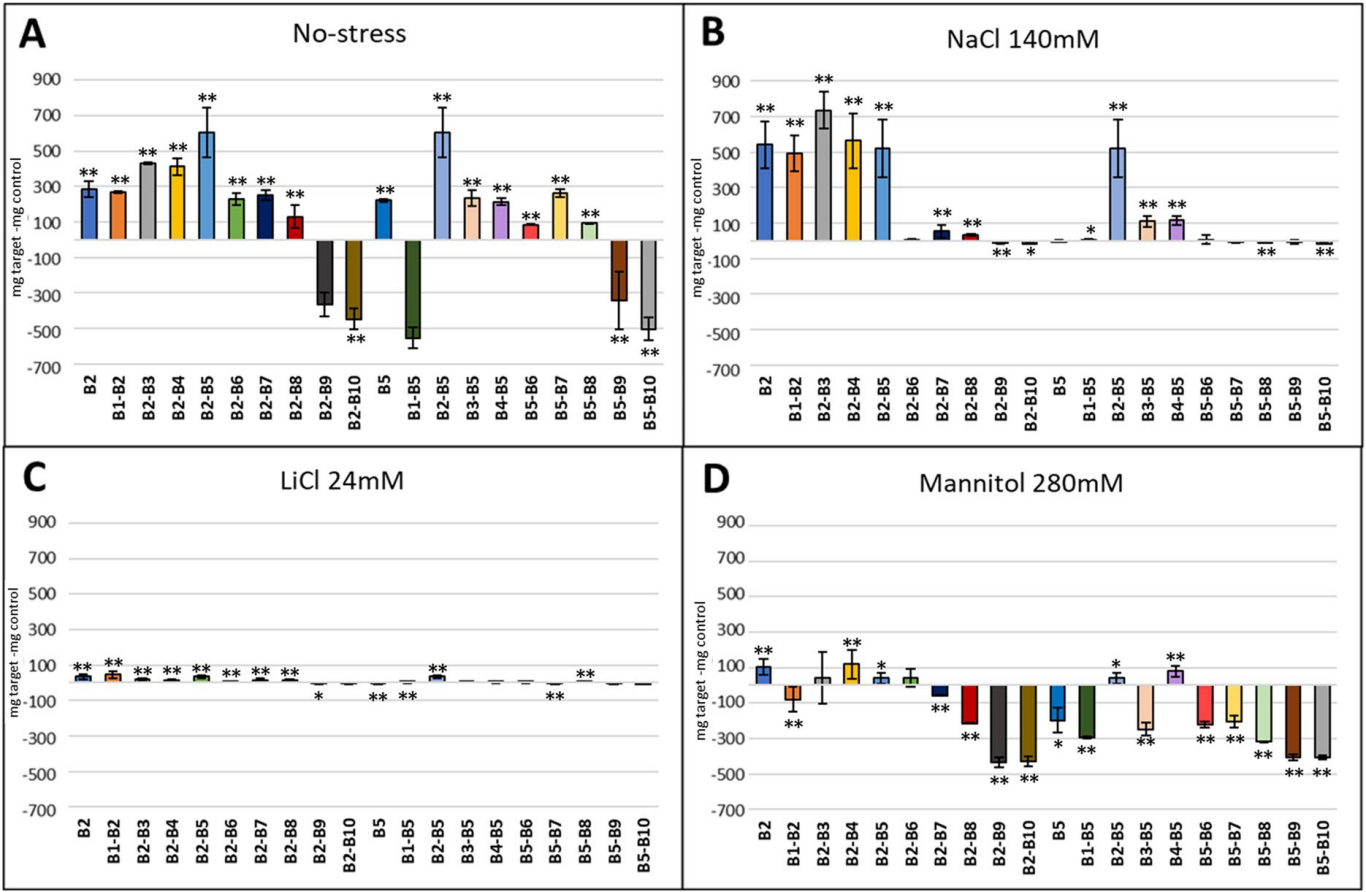
Effect of binary combinations on fresh weight of *A. thaliana* Col-0 WT under **A** control conditions (MS without stress) and in the presence of abiotic stress conditions: **B** NaCl 140 mM, **C** LiCl 24 mM, and **D** mannitol 280 mM. The optimal concentration of the natural extracts in *Arabidopsis* early growth was a concentration of 400 µg/ml (v/v) for B2 and B8, while the rest of the products showed an optimal concentration of 200 µg/ml (v/v). The X-axis indicates the tested product while the Y-axis represents the difference in fresh weight compared to the control sample without biostimulant. Statistical data from three experiments (n = 30 for each experiment) are presented. The bars represent a standard error. *p < 0.05 by Student’s tests for increased early development compared to the control; **p < 0.01 by Student’s tests for increased early development compared to the control

**Fig. 8:**
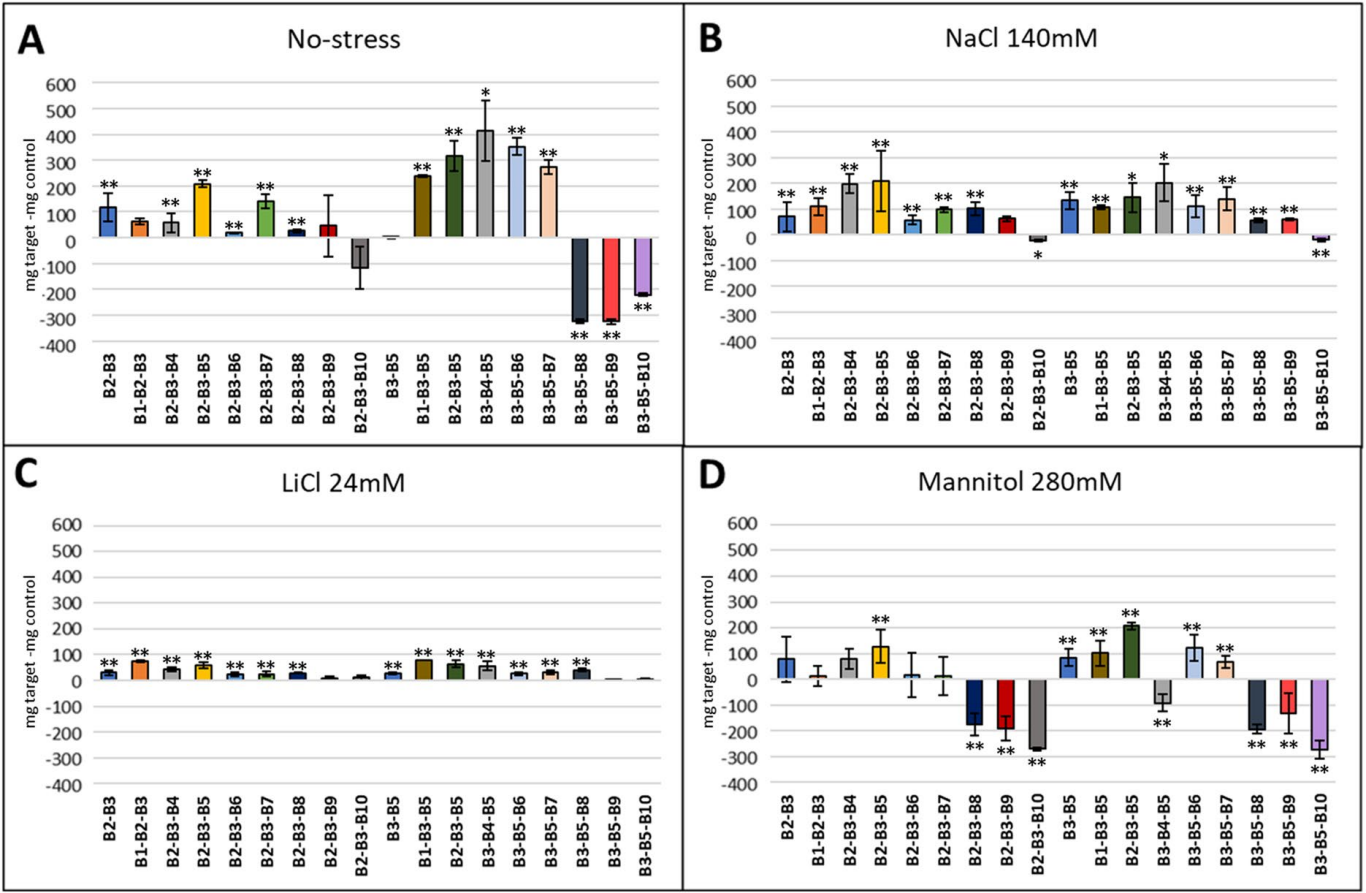
Effect of ternary combinations on fresh weight of *A. thaliana* Col-0 WT under **A** control conditions (MS without stress) and in the presence of abiotic stress conditions: **B** NaCl 140 mM, **C** LiCl 24 mM, and **D** mannitol 280 mM. The optimal concentrations were 50 µg/ml (v/v) for most of the compounds, except for B2, B6 and B7, which showed an optimal concentration of 250, 100 and 150 µg/ml (v/v) respectively. The X-axis indicates the tested product while the Y-axis represents the difference in fresh weight compared to the control sample without biostimulant. Statistical data from three experiments (n = 30 for each experiment) are presented. The bars represent a standard error. *p < 0.05 by Student’s tests for increased early development compared to the control; **p < 0.01 by Student’s tests for increased early development compared to the control

**Fig. 9:**
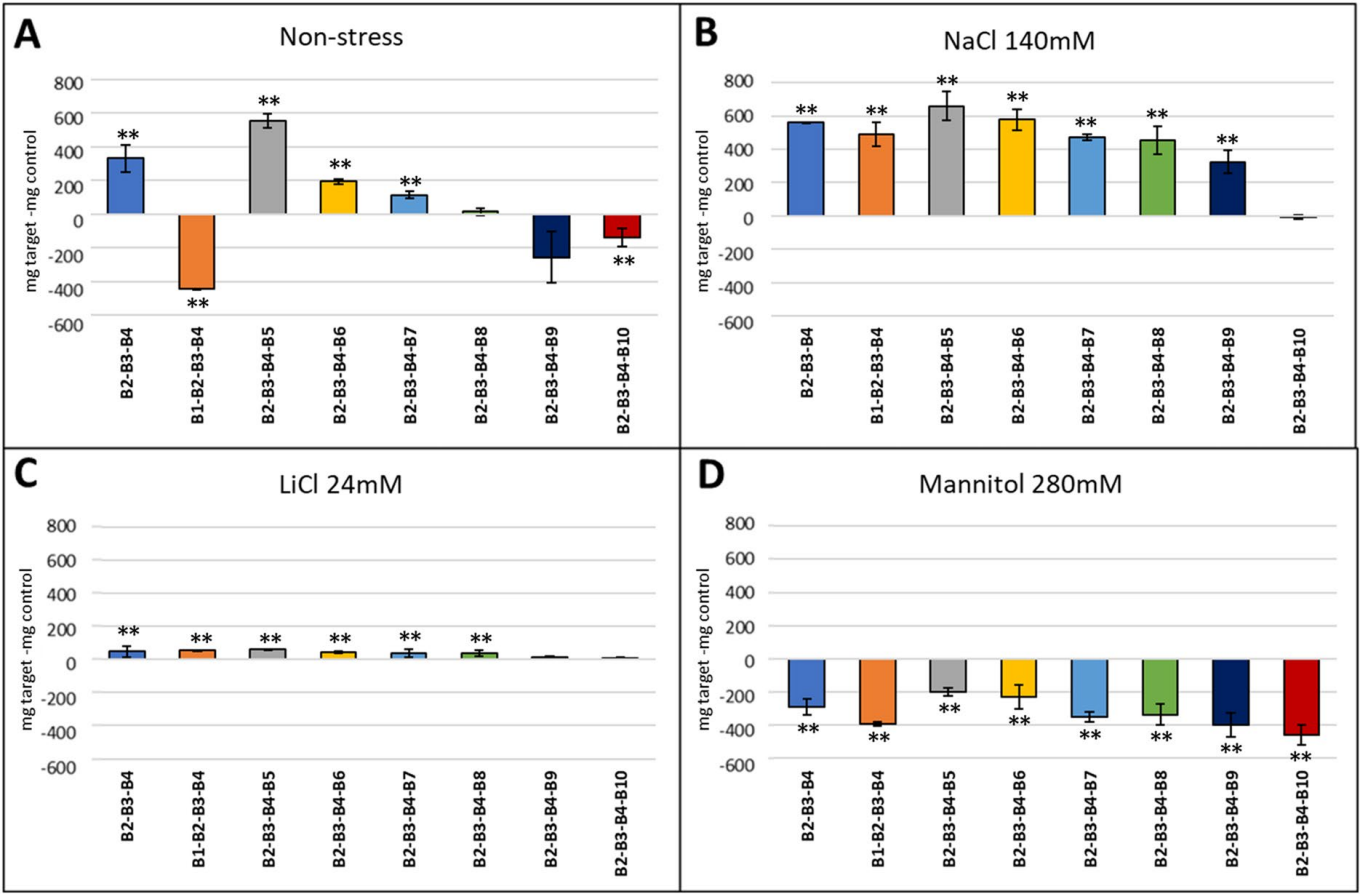
Effect of quaternary combinations on fresh weight of *A. thaliana* Col-0 WT under **A** control conditions (MS without stress) and in the presence of abiotic stress conditions: **B** NaCl 140 mM, **C** LiCl 24 mM, and **D** mannitol 280 mM. The optimal concentrations were 50 µg/ml (v/v) for most of the compounds, except for B2, B6 and B7, which showed an optimal concentration of 250, 100 and 150 µg/ml (v/v) respectively. The X-axis indicates the tested product while the Y-axis represents the difference in fresh weight compared to the control sample without biostimulant. Statistical data from three experiments (n = 30 for each experiment) are presented. The bars represent a standard error. *p < 0.05 by Student’s tests for increased early development compared to the control; **p < 0.01 by Student’s tests for increased early development compared to the control

The individual evaluation of the natural extracts in the presence of salt stress (140 mM NaCl and 24 mM LiCl), osmotic stress (280 mM Mannitol) and normal conditions showed that the compounds with the greatest stimulating effect were B2 and B5 (Fig. 6). These two compounds were selected to carry out the binary combinations. The combinations B2–B3, B2–B4 and B2–B5 presented better results (Fig. 7). In the case of ternary combinations, those combinations that contained compounds B2, B3, B4 and B5 exhibited better results, especially in the presence of salt stress (Fig. 8). We further investigated whether the combination of these four extracts was conferring any kind of effect. Under normal conditions and under salt stress the combination B2–B3–B4–B5 presented a positive effect (Fig. 9), but in osmotic stress no quaternary combination had a positive effect with respect to the control. Plants in the flowering stage were only observed in the ternary and quaternary combinations under normal conditions, but there was no flowering in those plants grown under abiotic stress. The combinations that showed flowering were those composed of products B1, B2, B3, B4, B5 and B8, but the best combination with the highest number of flowers was B2–B3–B4 (data not shown).

## Discussion

The combination of different natural extracts of plant origin can have a beneficial synergistic effect on plant growth under normal or abiotic stress conditions, but there is a very limited number of descriptions in the literature of methods suitable to find such synergies. We have further developed our previous method designed [19] and applied it in a combinatorial manner. This methodology can be performed in a short period (6–12 months) at a low cost and without the requirement of field tests.

By doing this, we take advantage of the fact that the model organisms that we are using (Baker’s yeast and *A. thaliana*) grow fast and have low space requirements. These characteristics allow us to carry out a considerable number of assays to test different conditions in a short period of time and with limited use of space and reagents. There are many yeast-based molecular biology techniques that can be applied to study plants [24, 25]. Yeast is also a standard model to investigate plant stress responses, as many mechanisms are conserved in plants. In fact, there are plant genes able to confer stress tolerance in plants that were identified upon overexpression in yeast [26, 27]. We have shown previously that biostimulants with growth-promoting effects or stress-alleviating effects in plants, also have a similar effect in yeast [19]. The results presented in this report further confirm the idea that yeast is an excellent system for biostimulant identification and characterization, and in addition, it provides an excellent platform to evaluate synergies among them.

In this report, we have confirmed this observation: In the yeast screening, we detected the biostimulant effect under salt stress of products B1 to B7 and the deleterious or weak effects of B8 to B10. We have confirmed that B1 to B7 also have a biostimulant effects in plants under salt stress and that B9 and B10 also have a deleterious effect in Arabidopsis. B8 is the only product which had different effects in yeast vs. plants. B8 is a yucca extract that we show here to be toxic for yeast. Therefore its biostimulant effect is likely masked by its fungal toxicity. The antifungal activity of yucca extracts has been associated with the presence of saponins among other compounds [28]. One of the best performing products in yeast is product B3. As described, we performed all the assays on a blind test-basis. B3 is an extract of the seaweed knotted kelp (*Ascophyllum nodosum*). This extract is a well-characterized biostimulant [29, 30], therefore the fact that we have identified it as one of the top performers in both yeast and in plants constitutes an internal positive control and confirms the efficiency of the methodology presented in this report. We have shown previously that plant biostimulants also have growth-promoting properties in yeast [19]. Other natural extracts that stood out for their biostimulant effect were B2 and B4 (yeast extract and Licorice root extract, respectively). Several authors have demonstrated the biostimulant effect of these compounds [31–33]. In the case of yeast extract, it has previously been tested in combination with seaweed extract, showing a good effect on the development and quality of tomato fruits [34, 35].

Some authors suggest that the investigation on biostimulants should focus on efficacy and safety and that finding the mode of action at the molecular level is not a priority [36]. Our method could give some insights into the mechanism of action. A product presenting a clear effect in yeast and plants should be affecting a conserved mechanism of stress response. For instance, the increase of potassium uptake is a parameter known to counteract the effects of salt stress in plants and yeast [37]. So, it is likely that a mechanism based on increasing potassium uptake should be easily detected in our methodology. In fact, there are reports in the literature that describe biostimulants that increase potassium uptake [38]. Another important feature is that toxicity or adverse effects are easily detected at early stages, thus avoiding subsequent analysis and saving resources. In a field test protocol, the toxicity or a deleterious effect could only be observed after the experimental field is set, or even at the harvest stage.

The main finding of this study is that synergies among different natural product extracts can be found in a cheap and fast manner using the methodology herein described. Specifically, we have found that a combination of four extracts (B2, B3, B4 and B5), that is, Yeast extract, *A. nodosum* extract, Licorice root extract and White willow extract can increase plant growth under normal and salt stress conditions. Arriving to this conclusion using crop plants and field tests would have taken several years and required a substantial budget. In addition, this kind of study produces a large amount of quantitative information on the effect of different combinations under different kinds of stress. This allows for the preparation of different formulations with specific effects (under a given stress or under normal growth conditions) and the evaluation of the cost–benefit analysis, for instance, by determining which combination has the best effectivity at a lower cost. In these experiments we used known biostimulants as internal controls. Importantly, products such as B3 (*A. nodosum* extract), with proven effectiveness under field conditions in crop plants, were efficiently identified in our system [39]. Future studies will test the efficiency of the most effective combination identified by this method (B2, B3, B4, and B5) with crop plants in greenhouse and field conditions.

## Conclusions

By using this novel methodology, we have been able to evaluate the biostimulant effect of 10 different products and their synergistic effects in a short time with low budget and technical requirements. This method permits fast screening of different products and the preparation of novel formulations with enhanced biostimulant effects. In addition, the described methodology also enables fast and cheap screening for previously undescribed synergies among bioactive compounds and also antagonistic interactions with deleterious biological effects. One of the major advantages is that these products can be discarded at early stages, avoiding subsequent tests and saving costs. This report provides a novel and convenient methodology useful for designing novel biostimulants.

## Supplementary Information

**Additional file 1: Figure S1.** Early growth assays of *Arabidopsis thaliana* grown in six-well Cellstar plates.

**Additional file 2: Figure S2.** Effect of quaternary combinations of natural extracts on *S. cerevisiae* growth under control conditions (YPD without stress), saline (NaCl and LiCl), osmotic (Sorbitol) and temperature (10 °C and 37 °C) stress.

## Data Availability

All data generated or analysed during this study are included in this published article.
