# Supplementary figures and images for: A fast method to evaluate in a combinatorial manner the synergistic effect of different biostimulants for promoting growth or tolerance against abiotic stress
Source: Plant Methods. 2022 Sep 15;18:111. doi: 10.1186/s13007-022-00943-6 (PMC9479394; doi:10.1186/s13007-022-00943-6)

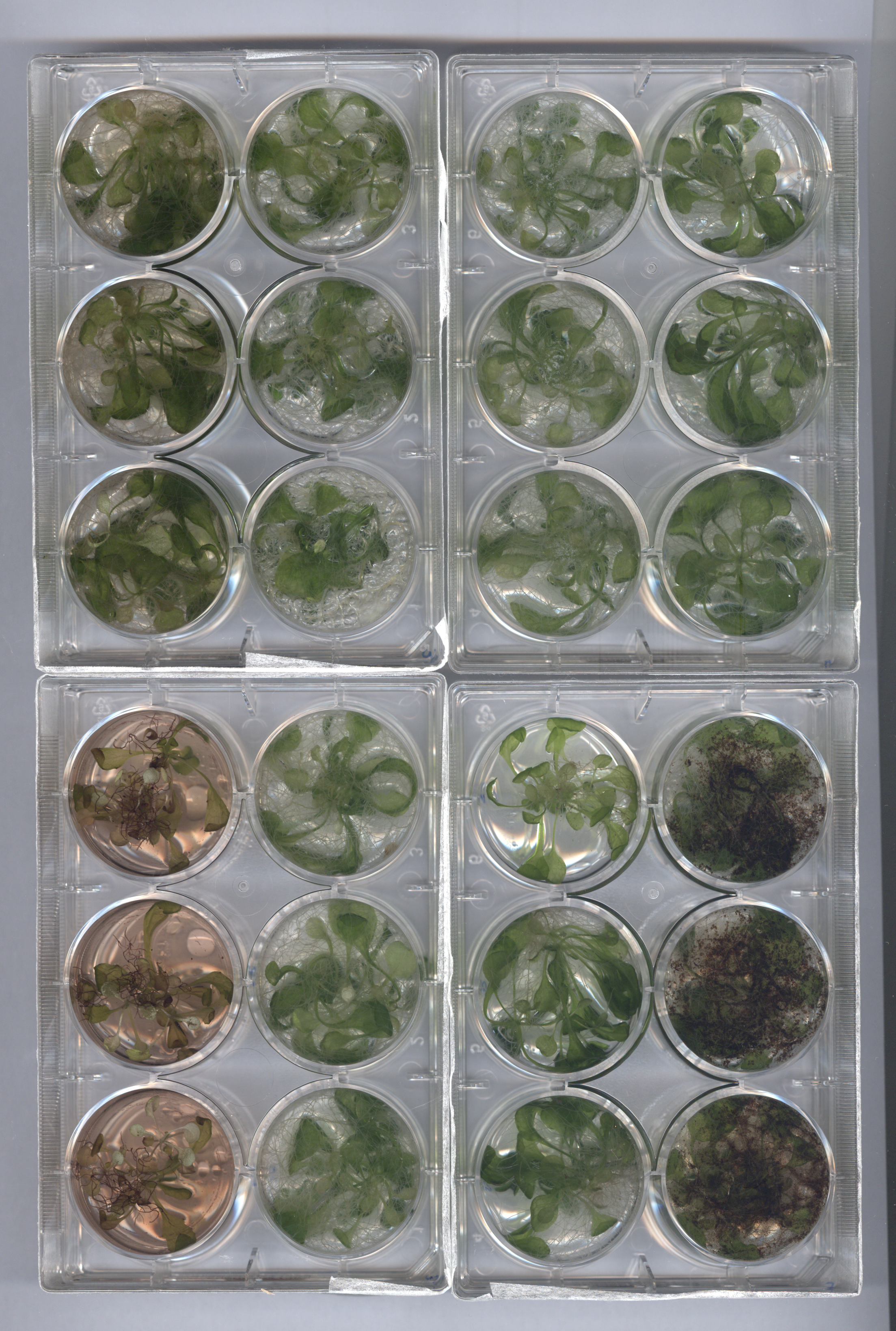

Supplement: Supplementary file 1 — Additional file 1: Figure S1. Early growth assays of Arabidopsis thaliana grown in six-well Cellstar plates. [file 13007_2022_943_MOESM1_ESM.tiff]

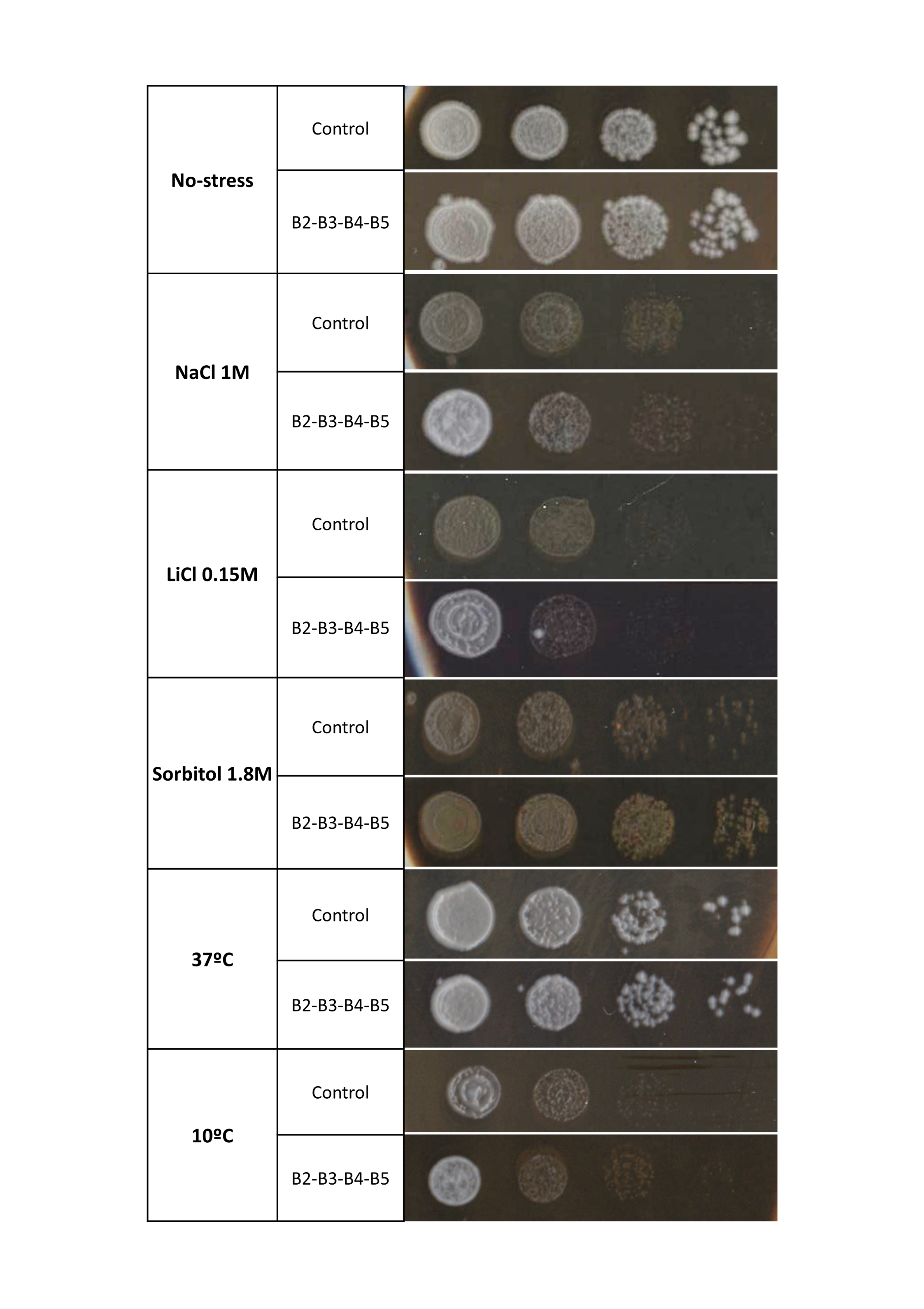

Supplement: Supplementary file 2 — Additional file 2: Figure S2. Effect of quaternary combinations of natural extracts on S. cerevisiae growth under control conditions (YPD without stress), saline (NaCl and LiCl), osmotic (Sorbitol) and temperature (10 °C and 37 °C) stress. [file 13007_2022_943_MOESM2_ESM.tif]
